# Vitamin D and Inflammatory Cytokines in Healthy and Preeclamptic Pregnancies

**DOI:** 10.3390/nu7085293

**Published:** 2015-08-04

**Authors:** David Barrera, Lorenza Díaz, Nancy Noyola-Martínez, Ali Halhali

**Affiliations:** Department of Reproductive Biology, Instituto Nacional de Ciencias Médicas y Nutrición Salvador Zubirán, Vasco de Quiroga No. 15, Tlalpan, México D.F. 14000, Mexico; E-Mails: barrera1912@gmail.com (D.B.); lorenzadiaz@gmail.com (L.D.); biolirio_12@hotmail.com (N.N.-M.)

**Keywords:** vitamin D, inflammatory cytokines, pregnancy, preeclampsia

## Abstract

Preeclampsia is a pregnancy disease characterized by hypertension and proteinuria. Among several disorders, the imbalance of inflammatory cytokines and the alteration of vitamin D metabolism have been reported in preeclampsia. The effects of calcitriol upon inflammatory cytokines has been demonstrated. In healthy pregnant women there is a shift toward a Th2 cytokine profile, which is necessary for an adequate pregnancy outcome. As compared with normal pregnancy, high pro-inflammatory and low anti-inflammatory cytokine levels have been observed in preeclamptic women. Preeclampsia has been associated with low calcitriol levels and vitamin D deficiency is correlated with a higher risk of the development of this disease. It has been demonstrated that placenta is a source as well as the target of calcitriol and cytokines and placental dysfunction has been associated with preeclampsia. Therefore, the present manuscript includes a review about serum calcitriol levels in non-pregnant, pregnant, and preeclamptic women as well as a review on the fetoplacental vitamin D metabolism in healthy and preeclamptic pregnancies. In addition, circulating and fetoplacental inflammatory cytokines in healthy and preeclamptic pregnancies are reviewed. Finally, the effects of calcitriol upon placental pro-inflammatory cytokines are also explored. In conclusion, maternal and placental calcitriol levels are low in preeclampsia which may explain, at least in part, high pro-inflammatory cytokine levels in this disease.

## 1. Introduction

Preeclampsia is a pregnancy disease characterized by hypertension and proteinuria [1]. Among several disorders, the alteration of vitamin D metabolism and the imbalance of inflammatory cytokines have been reported in preeclampsia [2,3,4,5,6,7,8,9,10]. In addition to its calcium effects, calcitriol, the active form of vitamin D, exerts non-calcemic actions [11]. A relationship between calcitriol and inflammatory cytokines has been demonstrated [12,13,14,15]. Since preeclampsia is associated with placental dysfunction, the present review includes the findings of several studies related to calcitriol and cytokines in healthy and preeclamptic pregnancies.

## 2. Calcitriol in Non-Pregnant and Pregnant Women

In non-pregnant women, calcitriol (1,25-dihydroxivitamin D) is synthesized in the kidney from calcidiol (25-hydroxivitamin D) through the action of the 25-hydroxivitamin D-1alpha-hydroxylase (CYP27B1). Calcidiol is the product of the hydroxylation in the liver of cholecalciferol (vitamin D_3_ from an animal source) and ergocalciferol (vitamin D_2_ from a vegetal/fungi source). This hydroxylation is catalyzed by the vitamin D-25-hydroxylase (CYP27A1, CYP2R1, CYP2J2, or CYP3A4). It is important to indicate that the human body receives the major amount of vitamin D from cutaneous synthesis of cholecalciferol, whose precursor is 7-dehydrocholesterol [11]. Indeed, it has been reported [16] that skin produces cholecalciferol photochemically from its precursor, which is present in the epidermis, by the action of sunlight or artificial ultraviolet light that converts the 7-dehydrocholesterol to previtamin D, which is in turn thermally isomerized to vitamin D. Renal calcitriol synthesis is tightly adjusted to calcium and phosphate needs [11]. In addition to these two nutrients, renal calcitriol production is regulated by parathyroid hormone (PTH) [17]. When circulating calcitriol levels are high, the excess of this secosteroid is catabolized to inactive products by the 25-hydroxivitamin D-24-hydroxilase (CYP24A1) [11]. Vitamin D receptor (VDR) is largely distributed in the body and mediates calcitriol effects [11,16]. In addition to its calcemic effects, it has been demonstrated that this hormone is involved in non-calcemic actions. Indeed, the role of vitamin D has been described in different conditions including immune and autoimmune diseases [18]. During pregnancy, the role of calcitriol in the regulation of the immune function at the maternal-fetal interface has been also reviewed [19].

During the last trimester of pregnancy, most of the skeletal fetal bone mineralization takes place [20]. At this time period, maternal calcium requirements increase. Interestingly, maternal circulating calcitriol levels increase in parallel fashion, resulting, at least in part, in higher calcium intestinal absorption that covers the maternal and fetal needs of this nutrient [21]. As previously reported, the maternal calcitriol level rises during the first trimester and then plateaus until term [20,22]. However, in our and other longitudinal studies [23,24], it has been shown that maternal calcitriol concentrations keep increasing in the second and third trimesters of pregnancy. Overall, these observations may suggest that increased calcitriol in the first trimester could be related mainly to the non-calcemic effects of this hormone, and its increase in the second part of pregnancy could explain, at least in part, higher calcium intestinal absorption according to the fetal requirements of this nutrient. Interestingly, Hollis *et al.* [25] have shown a significant association between circulating levels of calcitriol and calcidiol in pregnant women who received 400, 2000, or 4000 international units (IU) vitamin D/day from 12–16 weeks of gestation until delivery. However, this association has been observed only with calcidiol concentrations under 40 ng/mL. Indeed, increased calcidiol levels above this concentration have not been associated with significantly higher calcitriol. On the other hand, a calcidiol concentration of 40 ng/mL has been considered by the authors of this study to be required to reach an optimum concentration of calcitriol [25], while calcidiol levels under 40 ng/mL were considered vitamin D insufficient or deficient. When observed, this association indicated that calcitriol production is calcidiol-dependent during pregnancy. In contrast, an inverse association between calcidiol and calcitriol are observed in non-pregnant subjects with vitamin D deficiency and insufficiency [26]. Therefore, calcitriol levels are differentially controlled in pregnant and non-pregnant women in insufficient and deficient conditions. In non-vitamin D supplemented pregnant women, maternal calcitriol concentrations increase without significant changes in serum calcidiol levels [27].

Increased calcitriol levels during pregnancy could not be attributed only to a higher circulating PTH concentration since we have shown a significant increase in plasma calcitriol levels in both thyroparathyroidectomized and control pregnant rats, indicating that increased plasma calcitriol levels in pregnancy are PTH-independent [28]. Similarly, in pregnant women, the increase of this secosteroid has not been associated with PTH [23,29]. In addition, serum calcitriol levels increased two- to three-fold in pregnant women with pseudohypoparathyroidism while serum PTH was decreased by about 50% in these patients [30]. It is possible that insulin-like growth factor I (IGF-I) and parathyroid hormone-related peptide (PTHrP) could be potential stimulators of calcitriol synthesis during pregnancy. Indeed, both maternal circulating IGF-I and PTHrP increase during pregnancy [23,24] and correlation studies have shown significant associations between calcitriol and IGF-I [24] and PTHrP [23]. Interestingly, IGF-I has been suggested as an additional regulator of vitamin D metabolism since this growth factor stimulates calcitriol synthesis in mouse kidney [31] and human placenta [32]. Regarding PTHrP, it has been observed that administration of this peptide to mice was associated with increased serum calcitriol levels [33] and renal CYP27B1 activity [34]. In healthy human volunteers, PTHrP infusion resulted in an increase of not only calcitriol [35,36] but also IGF-I levels [35]. These findings suggest that IGF-I and PTHrP may have an important role in vitamin D metabolism during pregnancy which deserves to be further investigated.

## 3. Calcitriol in Preeclamptic Pregnancy

### 3.1. Association between Calcitriol and Hypocalciuria in Preeclampsia

Several alterations of calcium metabolism have been observed in preeclampsia, and the most common of them is hypocalciuria, which has even been suggested as an early predictor of the development of the disease [37,38]. The pathophysiological mechanisms involved in low urinary calcium excretion are still unclear. Thus, several studies have been done in order to investigate if hypocalciuria results from an alteration of vitamin D metabolism. Taufield *et al.* [38] were the first researchers who observed that preeclamptic patients have significantly lower 24-hour urinary total calcium excretion as compared with normotensive pregnant women, without significant differences in circulating calcitriol levels between the groups [38]. However, results from our studies [3,4,5,39] and other laboratories [2,6] showed that maternal serum levels of calcitriol are lower in preeclampsia. In our study [3], urinary total calcium excretion correlated significantly and positively with maternal serum calcitriol levels in both normotensive pregnant women and preeclamptic patients. In another study, we have shown that fractional urinary calcium excretion is significantly lower in preeclamptic women than in normotensive pregnant women [4]. Circulating calcitriol levels correlated significantly and positively with both total and fractional urinary calcium excretion in normotensive pregnant women [4]. In the preeclamptic group, serum calcitriol levels have been significantly associated with only total urinary calcium excretion [4]. Our finding of a positive association between renal calcium excretion and serum calcitriol suggests that low calcitriol levels in preeclampsia may contribute to a complex mechanism leading to hypocalciuria.

### 3.2. Alteration of Vitamin D Metabolism in Preeclampsia

The observation of abnormal vitamin D metabolism in preeclampsia has been reported by August *et al.* [2], who demonstrated that maternal calcitriol levels were significantly lower in preeclamptic women compared to normotensive pregnant women. In the same year, Seely *et al.* [6] have shown a similar observation since calcitriol levels were significantly lower in preeclamptic women than in normotensive pregnant controls without significant changes in calcidiol concentrations between groups. In our laboratory, we have determined circulating levels of calcitriol in preeclamptic and normotensive pregnant women and our data were in accordance with those described above since the preeclamptic group had significantly lower levels of this secoesteroid [3,4,5,39]. Decreased maternal calcitriol levels in preeclampsia have been observed at the moment of the diagnosis of the disease in the third trimester of pregnancy. However, in a longitudinal study, no significant changes in circulating calcitriol levels were observed between normotensive pregnant women and those who later developed preeclampsia [24]. Nevertheless, decreased maternal calcitriol levels may be observed early in pregnant women with a risk of preeclampsia development and who present vitamin D insufficiency or deficiency [25].

The cause of decreased calcitriol levels in preeclampsia is still unknown since classical inhibitors such as increased serum calcium or phosphate levels are not found in preeclamptic patients [3,4,5,6,38,40,41] and stimulators such as circulating PTH are not significantly different as compared with healthy pregnant women [3,4,5,38,40,41]. However, circulating levels of IGF-I have been found lower in preeclamptic women [3,4,5,39,42,43,44,45,46,47,48,49]. Furthermore, low placental IGF-I expression [47,50] and levels [39] have been found in preeclampsia. As shown for IGF-I, maternal circulating PTHrP levels have been found lower in preeclamptic women [51,52]. Therefore, low IGF-I and PTHrP may explain, at least in part, the decreased calcitriol levels observed in preeclampsia. However, additional studies are needed to ascertain this assumption.

Calcidiol, considered the vitamin D status marker, has been also studied in preeclampsia. Maternal serum calcidiol levels have been found to be lower in pregnant women who subsequently developed preeclampsia and in preeclamptic women with a diagnosis of early-onset severe preeclampsia [51,52,53,54,55,56,57,58,59,60,61,62,63,64,65,66,67,68,69]. However, other studies have not a found significant association between calcidiol levels and preeclampsia [70,71,72,73]. Since vitamin D status is influenced by sunlight exposure [74], Lechtermann *et al.* [75] studied the influence of the season upon maternal vitamin D status and they found that preeclamptic women have significantly lower serum calcidiol levels only in summer, while their calcitriol concentrations were significantly lower only in winter. It is noteworthy that the concentration of these two vitamin D metabolites have not been found significantly different in winter or summer, respectively, between healthy and preeclamptic pregnancies. It is possible that these findings may explain the controversy about the existence of an association between calcidiol concentration and preeclampsia development. As mentioned above, low calcitriol in maternal serum from preeclamptic women has been demonstrated in several studies. Therefore, the observation of a significant decrease in circulating levels of this secosteroid only in winter deserves to be confirmed by future investigations. On the other hand, several studies have found that vitamin D deficiency or insufficiency may represent a risk factor for preeclampsia development. Therefore, vitamin D supplementation during pregnancy has been suggested as a beneficial strategy to reduce the incidence of different diseases including preeclampsia [57,76,77]. The aim of vitamin D supplementation is to reach a circulating calcidiol level of 30 ng/mL or more without exceeding 150 ng/mL in order to avoid the risk of toxicity. This strategy is of importance for pregnant women with a vitamin D status diagnosed as insufficient or deficient (below 30 ng/mL and 20 ng/mL, respectively). However, Hollis *et al.* [25] estimated that circulating calcidiol levels of 40 ng/mL are required to optimize calcitriol production during pregnancy. In addition, calcium supplementation may be recommended for pregnant women with a dietary calcium intake below the requirement since several studies have demonstrated that this nutrient significantly reduces the risk of preeclampsia development [78].

### 3.3. Fetoplacental Synthesis of Calcitriol in Pregnancy

Calcitriol synthesis has been demonstrated in decidua and placenta, tissues of maternal and fetal origin, respectively. Indeed, human decidual cells have been considered an extrarenal source of calcitriol since it has been reported that the presence of 6 nM of tritiated calcidiol was readily hydroxylated, yielding a compound that was able to bind to a specific rachitic chick receptor and had a mass spectrum identical to and comigrated with authentic calcitriol [79]. On the other hand, the data of the study done by Weisman *et al.* [80] showed that vitamin D and calcidiol cross the placenta and enter the fetus. In addition, the presence of calcitriol has been observed in the maternal serum of nephrectomized pregnant rats and the authors have suggested that the fetal portion of the fetoplacental unit is the most likely site of calcitriol production [80]. Alternately, the study of Gray *et al.* [81] done on vitamin D deficient pregnant and non-pregnant rats demonstrated that nephrectomy prevented the conversion of calcidiol to calcitriol in non-pregnant rats, while reducing but not abolishing the formation of calcitriol from its precursor in pregnant rats. In another study, Weisman *et al.* [82] have demonstrated for the first time that human placenta is able to synthesize calcitriol, which indicates the extrarenal synthesis of this hormone in pregnant women. In addition, it has been demonstrated that human placenta expresses the VDR [24,83], reinforcing the plausibility of calcitriol effects in this organ. Thus, the researchers interested in this area used human placenta as a biological model for the study of vitamin D metabolism and also as a target of calcitriol. In human placenta, Zerwekh and Breslau [84] demonstrated that CYP27B1 activity was significant in trophoblasts. Hollis *et al.* [85] have shown calcitriol synthesis not only in mitochondria but also in microsomes isolated from human trophoblasts and suggested that placental production of this secosteroid was not dependent on the presence and activity of CYP27B1, therefore postulating the possibility that calcitriol synthesis in this tissue may result from the insertion of oxygen at carbon 1 of calcidiol by a free radical chemistry mechanism. However, in human syncytiotrophoblast cell cultures, we have demonstrated that the presence of cycloheximide significantly inhibited the stimulatory effect of IGF-I upon calcitriol synthesis, which indicated the participation of a protein enzyme in the biotransformation of calcidiol into calcitriol [32]. Later, we identified a CYP27B1 gene transcription product in cultures of human syncytiotrophoblast cells [86]. To date, several researchers have consistently observed calcitriol synthesis in human placenta [87,88]. Overall, both decidua and trophoblasts contribute to calcitriol production during pregnancy.

### 3.4. Placental Vitamin D Metabolism in Normotensive and Preeclamptic Pregnancies

**Figure 1:**
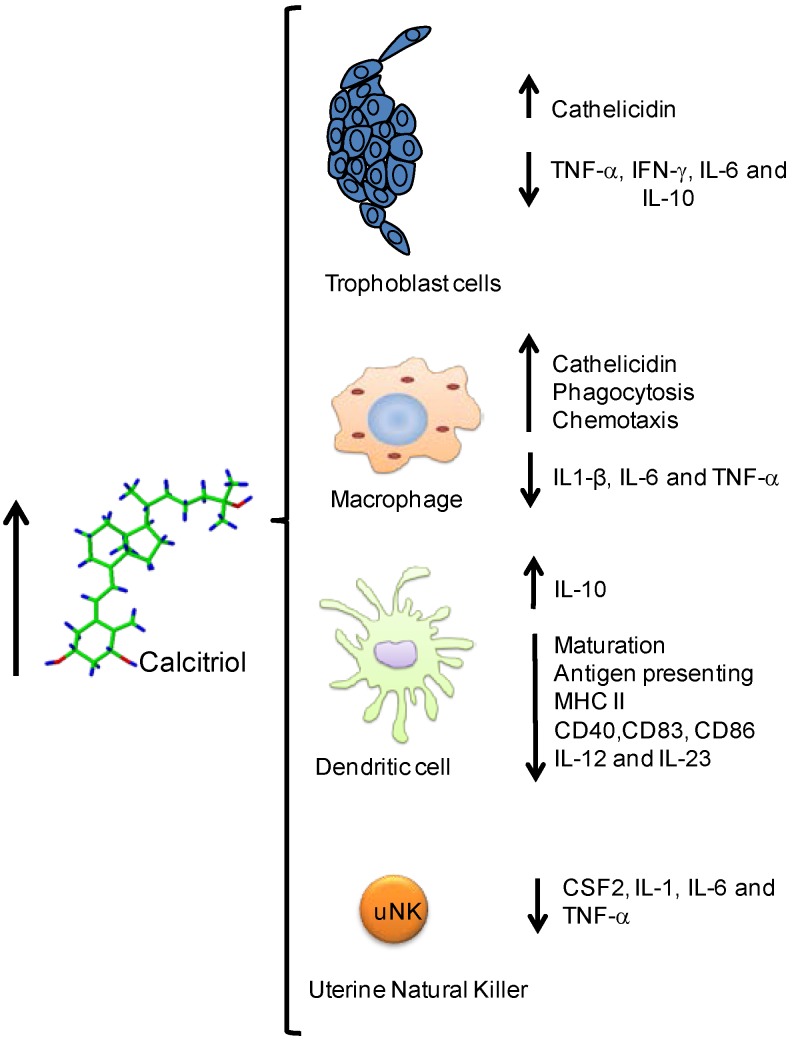
Calcitriol effects upon the immune system during pregnancy. Calcitriol regulates several components of the immune system at the systemic and fetoplacental compartments both in immune cells (macrophage, dendritic, uterine natural killer, and lymphocytic) and non-immune cells (trophoblast) leading changes toward a Th2 profile.

Although low calcitriol concentrations have been found in maternal and umbilical cord compartments from preeclamptic women as compared with normotensive pregnant women [2,3,4,5,6,39], the results of human placental CYP27B1 expression are controversial, depending on the biological material used. Indeed, when CYP27B1 has been studied in syncytiotrophoblast cells in culture, our laboratory has demonstrated low gene expression and activity of this enzyme in preeclampsia [89]. In addition, we have demonstrated that calcitriol was significantly lower in preeclampsia in human placental homogenates obtained from cotyledons [39]. In contrast, in whole human placental tissues, the expression of CYP27B1 mRNA [90] and protein [91] have been found higher in preeclampsia. Decreased placental calcitriol levels seen in preeclampsia may be due to the higher degradation of this secosteroid as a consequence of increased CYP24A1 expression that has been found in preeclampsia [91]. High CYP24A1 expression could be explained by the stimulatory effect of tumor necrosis factor (TNF)-α upon this catabolic enzyme as observed in human cultured trophoblast cells [92,93]. However, it has been observed that the promoter of CYP24A1 is methylated in normal human placenta, a process resulting in the decreased expression of the enzyme, and therefore preventing calcitriol degradation [94]. This observation deserves to be investigated in placentas obtained from preeclamptic women in order to establish if the promoter of CYP24A1 is differentially methylated in this disease in which the milieu is different to that observed in normal pregnancy. In summary, further investigations are needed in order to know if the placental calcitriol clearance is altered in preeclampsia. However, the finding of decreased placental calcitriol synthesis seen in preeclampsia [39,89] may be associated with several alterations of placental functions. Indeed, it has been demonstrated that calcitriol regulated placental human chorionic gonadotropin expression [95], placental steroid hormone secretion [96], placental calbindins expression [97], and placental invasion, as indicated by the increased secretion of matrix prometaloproteinases 2 and 9 by human extravillous trophoblasts [98]. In addition, calcitriol promoted vascular endothelial growth factor and the antioxidant CuZn-superoxide dismutase expression in endothelial cells [99], and reversed the adverse effects of preeclampsia serum or conditioned medium from hypoxic placenta on endothelial colony-forming cells' capillary tube formation and migration [100]. Furthermore, calcitriol has been considered a regulator of placental inflammatory cytokines [93,101,102] (Figure 1) and cytokines have been able to regulate placental CYP27B1 and CYP24A1 expression [92].

It has been recognized that preeclampsia development occurs in two stages [103]. An early preclinical stage I occurs during the first half of pregnancy, in which placental invasion and endothelial function are altered, and stage II occurs where the clinical signs such as hypertension and proteinuria are diagnosed after 20 weeks of pregnancy and where dysfunctional maternal endothelium and maternal systemic inflammation occur [103]. Tamblyin *et al.* [19] have published an interesting review related to the importance of the immunological role of vitamin D at the materno-fetal interface, particularly in the decidua, for fetal-maternal immune tolerance at phase I of preeclampsia and for the prevention of adverse events during the course of the whole pregnancy. However, the role of placenta as a source and target of cytokines has been also documented [104]. Therefore, the remaining review will be focused on circulating cytokine levels as well as fetoplacental cytokine expression in healthy and preeclamptic pregnancies. In addition, the effects of calcitriol upon cytokines in human placenta are also reviewed.

## 4. Circulating Cytokines in Healthy and Preeclamptic Pregnancies

Pregnancy is a unique condition where an organism with a different antigenic profile coexists peacefully while developing inside the mother in a tolerogenic environment until birth. The survival of the “fetal allograft” in mammalian pregnancies has long been considered a paradox (fetal antigens are recognized as foreign by the mother, yet the fetus continues to grow unrejected); however, it is much more complicated than just the simple nurturing and acceptance of an allograft [105]. Indeed, maintenance of pregnancy also implies a complex communication network between fetal and maternal tissues aimed to prevent excessive invasion of the maternal uterine wall while providing the fetus a quiescent environment. All these processes are undertaken by different types of immune maternal and fetal cells such as uterine natural killer cells (NK), dendritic cells, T cells, and macrophages, all of which are of particular importance at the feto-maternal interface. Placental cells such as trophoblast and decidual cells also contribute to this process. In order to sustain pregnancy by regulating the processes described above, immune and placental cells produce different molecules including growth factors and hormones as well as cytokines and chemokines. In particular, the cytokine profile during pregnancy has been a matter of several studies, given that its deregulation may endanger pregnancy, as seen in inflammatory pathologies such as preeclampsia (Table 1). During pregnancy, the placenta, a temporal organ of fetal origin, governs many of the immune features of the mother. Therefore, any alteration of placental regulatory factors may also abnormally systemically impact the mother’s immune profile.

**Table 1:** Inflammatory cytokines in preeclampsia.

Cytokines in Preeclamptic Pregnancies	References
Genetic polymorphisms of TNF-α and IL-1 result in increased levels of these pro-inflammatory cytokines and are associated with preeclampsia.	[106]
Serum and placental levels of pro-inflammatory IL-18 are increased in preeclampsia.	[107]
The ratios IL-2/IL-10 and TNF-α/IL-10 in maternal serum are higher in preeclampsia than in normal pregnancy.	[108]
Pro-inflammatory cytokines IL-6 and IL-8 are increased in maternal serum from women with preeclampsia as compared with normal pregnancy.	[109]
Serum levels of pro-inflammatory IL-15 and IL-16 are significantly higher in preeclampsia than in normal pregnancy.	[110]
Gene expression of anti-inflammatory IL-4 is low in preeclampsia.	[111]
In preeclampsia, the pro-inflammatory TNF-α and IL-6 and C-reactive protein are higher compared to normal pregnancy.	[112]

The T helper (Th) 1 and Th2 lymphocytes are distinguished by their opposite pattern of cytokine production. Th1 cells preferentially produce interferon-γ (IFN-γ), IL-2, and TNF. The potent inflammatory cytokine TNF-α, which is involved in systemic inflammation and acute phase reaction, is primarily produced by activated macrophages, but also may be produced by Th1 cells, NK cells, and placental cells. On the other hand, Th2 cells produce IL-4, IL-5, IL-6, IL-9, IL-10, and IL-13. It is noteworthy that the potent immunosuppressive cytokine IL-10 may also be produced by Th1 cells, while IL-6 may act both as a pro-inflammatory and as an anti-inflammatory cytokine. Other important immunomodulatory cytokines are the members of the IL-1 family such as IL-1α and IL-1β, which possess strong pro-inflammatory effects, and transforming growth factor β (TGF-β), which mainly exerts immunosuppressive effects by blocking lymphocyte activation.

Several researchers have studied the variation of serum cytokine levels as pregnancy progresses. A longitudinal study including 45 normal pregnant women found that basal and stimulated levels of IFN-γ, TNF-α, IL-1β, and IL-6 decreased throughout gestation, while lipopolysaccharide-stimulated IL-10 expression increased as pregnancy progressed [113]. Nonetheless, decreased serum IL-10 in normal pregnancy compared to healthy non-pregnant women has also been described [114]. Other studies have analyzed the cytokine secretion profile of *in vitro* activated peripheral blood lymphocytes obtained from normal term pregnancies, finding that Th2 dominance is a feature of a successful pregnancy [115]. Saito *et al.* [116], by using flow cytometry, were the first to show that in normal pregnancy the percentage of Th1 cells was significantly lower in the third trimester, and the ratio of Th1:Th2 was also significantly lower in the second and third trimester, compared to non-pregnant subjects. In contrast, the percentage of Th1 cells and the ratio of Th1:Th2 in preeclampsia were significantly higher than in normal third-trimester pregnant women. Accordingly, the percentage of Th2 cells in preeclampsia was significantly lower than in the third trimester of normal pregnancy [116]. Similarly, Szarka *et al.* [114], by using multiplex suspension array, found that with the exception of serum IL-1β and TGFβ1, the levels of all inflammatory cytokines tested differed significantly among normal and preeclamptic pregnant women *versus* non-pregnant women. In particular, normal pregnancy was found to be characterized by a shift towards Th2-type immunity and the inhibition of cytotoxic Th1 immune responses, while preeclampsia was associated with an overall pro-inflammatory systemic environment. The authors attributed the Th2-type immunity in normal pregnancy to the relative abundance of circulating IL-18 over IL-12p70 and the relative deficiency of the bioactive IL-12p70 in relation to IL-12p40. Meanwhile, serum IL-12p70 levels were significantly higher in preeclamptic women as compared to healthy pregnant women [114].

The predominance of the type 2 immune response during normal pregnancy is understandable given the need of a pro-tolerogenic environment. Nevertheless, Holtan *et al.* [117] have recently suggested that the description of pregnancy in terms of type 2 immune responses may be an oversimplification, both in the overall description of key cytokines as well as in the temporal dynamics. This postulate was based on their analysis of the levels of different cytokines using a multiplex bead-based assay on serum from a small group of pregnant women from the first trimester through parturition. They found that as pregnancy progresses, a maternal shift away from a type 2-biased immune response and towards an inflammatory/counter-regulatory response was observed. In particular, this research group found that IL-1β, IL-6, IL-8, IL-12p70, IL-13, and IL-15 increased as pregnancy advanced. Similarly, Christian *et al.* [118] found a tendency for increased IL-6 levels across pregnancy and a significant rise in TNF-α from early and middle to later normal pregnancy. In accordance, Mor *et al.* [119] proposed to change the paradigm of pregnancy from an overall immunosuppressed state to a new one that takes into account fetal-maternal immune communication as well as the immunological response of the mother to microorganisms. In fact, in several studies, normal pregnancy has been found to be associated with an overall mild increase in both Th1 and Th2 cytokines [120,121,122,123,124,125,126].

On the other hand, even though preeclampsia pathophysiology remains far from clear, in general, the association of placental-derived factors such as inflammatory cytokines with the preeclampsia phenotype has been widely demonstrated and is commonly associated with the genesis of this complex syndrome. As mentioned above, the studies by Saito *et al.* [116] and Szarka *et al.* [114] consistently showed an overall pro-inflammatory systemic environment with increased Th1 cytokines. These observations have been supported by many other studies; for example, Conrad *et al.* [127] found that the median concentrations of plasma TNF-α and IL-6 were increased two-fold and three-fold in preeclamptic women compared to normal third-trimester pregnant women, respectively [127]. However, no differences in plasma IL-1β and IL-10 were found between groups [127]. Likewise, preeclamptic women in an Iranian study showed significantly higher serum levels of TNF-α and IL-15 but lower IL-10 levels in comparison with normotensive pregnant women [128]. Moreover, two recent systematic reviews and meta-analyses found maternal serum TNF-α, IL-6, and IL-10 concentrations all significantly higher in preeclamptic patients *versus* controls [129,130]. These findings might be explained in part by the fact that the three cytokines TNF-α, IL-6, and IL-10, which are normally down-regulated by calcitriol at the placental level [93,101], could be over-expressed due to the decreased placental production of calcitriol seen in placentas obtained from preeclamptic women [39,89].

In particular, IL-6 and TNF-α are important players contributing to the endothelial dysfunction observed in preeclampsia. Other authors have also found these two cytokines as well as TNF-α soluble receptors significantly higher in the serum of preeclamptic patients, compared with age- and gestation-matched controls in the third trimester of pregnancy [125,131,132,133].

## 5. Fetoplacental Cytokines in Healthy and Preeclamptic Pregnancies

**Figure 2:**
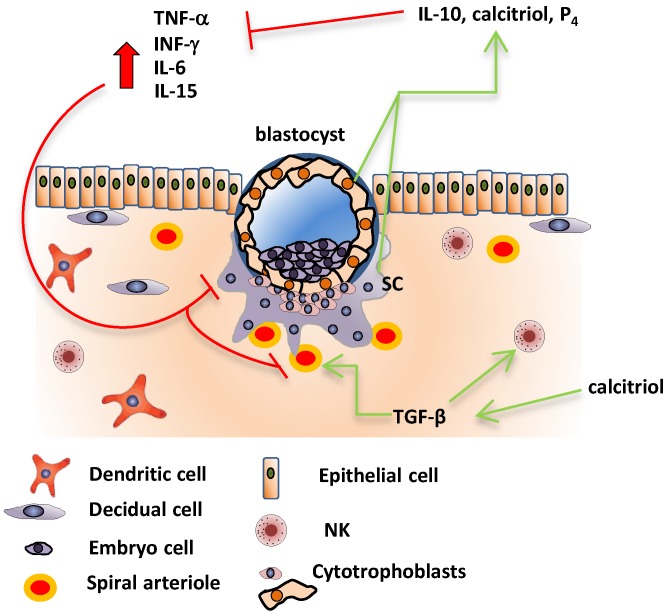
Cytokine profile during implantation. An early state of controlled inflammation at the feto-maternal interface is needed to promote adequate trophoblast invasion. TNF-α controls trophoblast growth and invasion of maternal spiral arteries, limiting excessive trophoblastic penetration. Another placental cytokine involved in this process is TGF-β, which interacts with its receptor endoglin in blood vessels and controls trophoblast invasion/penetration. TGF-β produced by macrophages interacts with NK cells, making them accept trophoblasts while avoiding them to kill fetal cells. In order to prevent excessive inflammation that could result in the rejection of the fetal allograft, calcitriol, IL-10, and progesterone (P_4_) produced by decidual cells, trophoblasts, and syncytiotrophoblasts act as anti-inflammatory factors modulating the immunological milieu. Calcitriol and IL-10 may also be produced by immune cells present in the feto-maternal interface. Calcitriol is also known to induce TGF-β.

In pregnancy, the immune system is not completely Th1 or Th2, but an active and cautiously modulated system that allows fetal development while carefully fighting infectious threats. This is especially important at the implantation site. For example, during implantation, high amounts of pro-inflammatory cytokines such as IL-6, IL-15, and TNF-α, as well as several chemokines, are detected [134]. This early state of controlled inflammation at the feto-maternal interface is needed to promote adequate trophoblast invasion [135,136] (Figure 2). TNF-α was reported to play a role for adequate trophoblast growth and invasion of maternal spiral arteries, and also in limiting excessive trophoblastic infiltration [137]. TNF-α is one of several decidua/trophoblast/immune cell-derived factors with the capacity to inhibit extravillous trophoblast invasion. Other placental cytokines involved in this process are TGF-β1, 2, and 3, which inhibit trophoblast invasion by a mechanism dependent on reduced protease activity, and interferon (INF)-γ, which works in concert with TNF-α [138,139].

**Figure 3:**
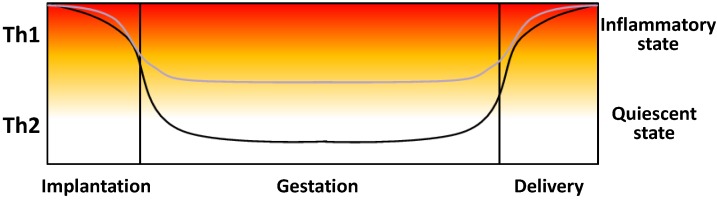
Schematic representation of the cytokine profile from implantation to parturition. The line in black represents a normal pregnancy, while the line in grey represents one complicated with preeclampsia. During implantation, high amounts of pro-inflammatory cytokines such as IL-6, IL-15, and TNF-α are needed so that trophoblasts can invade the maternal vascular and endometrial tissue, which results in the recruiting and activation of immune cells. Then, pregnancy continues with a peaceful immune profile, allowing the fetus to grow. Finally, for parturition to occur, reactivation of inflammation must take place. In preeclampsia, an overall pro-inflammatory systemic environment with increased Th1 cytokines has been observed in maternal serum, amniotic fluid, and umbilical serum.

Significant amounts of TNF-α, IL-6, and IL-10 have been detected in the normal maternal decidua and chorionic villi of human placenta, but IFN-γ is scarcely detected [140]. Mor *et al.* [119] clearly explain how inflammation is required during both implantation and parturition. Indeed, during implantation, the trophoblast actively struggles to invade and prevail by damaging the maternal vascular and endometrial tissue, resulting in the recruiting and activation of immune cells. Then, the second trimester of pregnancy takes place with a peaceful immune profile (anti-inflammatory), allowing the fetus to grow. Finally, for parturition to occur, the reactivation of inflammation must take place (Figure 3) [119].

However, it is also well recognized that exacerbated/perpetuated inflammation may lead to pregnancy complications such as spontaneous abortion, preterm labor, preterm rupture of membranes, and preeclampsia. Placental-derived inflammatory factors in abnormal proportions have been associated with the pathology of preeclampsia. For instance, amniotic fluid and umbilical serum TNF-α levels were found significantly higher in severe preeclamptic pregnancies than in normotensive controls [132,141]. Accordingly, placental TNF-α concentrations and mRNA expression are also increased [142]. On the other hand, IL-37 and IL-18BP, which are considered anti-inflammatory cytokines, have been found significantly up-regulated in placentas obtained from preeclamptic women compared to those from normal pregnancies [143]. This finding has been suggested to be a compensatory placental mechanism to prevent inflammatory damage to the mother [143].

Interestingly, TNF-α and IL-6 expression has been shown to be significantly higher in umbilical arteries and veins from preeclamptic women than controls [144]. Moreover, flow cytometry analysis of lymphocyte subsets isolated from third-trimester decidua showed an increased percentage of the CD3−/CD56+ CD16+, CD8+/CD28+, and a decreased percentage of CD3+, CD19+, CD4+/CD45RA+ lymphocytes in the samples of women with preeclampsia compared to controls. Meanwhile, the profile of secreted cytokines showed significantly higher IFN-γ and lower IL-6, IL-12, and IL-10 secretion [145]. As previously postulated by Sargent *et al.* [146], high INF-γ in placentas obtained from preeclamptic women raises some questions, given that normal placentas barely express this cytokine [146,147], which is a product of NK and T cells critical for fighting viral infections.

In a recent large-scale proteomic analysis, Wang and colleagues performed a comparative proteome profile of human placentas from normal and preeclamptic pregnancies using liquid chromatography-tandem mass spectrometry [148]. The data revealed that 243 peptides were significantly and differentially expressed between normal and preeclamptic placentas. Interestingly, one of the top three networks identified that differentially expressed proteins were those involved in immune system processes. In fact, 21 immunoregulatory proteins were found down-regulated in placentas obtained from preeclamptic women, which included interleukin-27-β and TGF-β together with its receptor CD105 (endoglin) [148]. Endoglin, which mediates TGF-β signaling by interacting with TGF-β receptors I and/or II, was down-regulated 3.5-fold compared to normal placentas. This might be highly relevant in the placenta, given that endoglin is strongly expressed in blood vessels. Its important proangiogenic role has been highlighted by studies showing that endoglin null mice die *in utero* as a result of impaired angiogenesis in the yolk sac [149,150]. It is noteworthy that a key pathogenic mechanism of preeclampsia is that the PE placenta shows impaired invasion of the uteroplacental arteries by extravillous trophoblasts, resulting in insufficient vessel remodeling and aberrant vascularization. Moreover, an excess in resident macrophages at the placental bed of preeclamptic women limits trophoblast invasion of spiral arteries through apoptosis mediated by the combined effect of TNF-α secretion and decreased tryptophan [151]. It is of note that polymorphisms in genes encoding for proteins in the TGF-β signaling pathway are associated with the severity of preeclampsia [152]. Therefore, the immune cell population that normally resides at the feto-maternal interface as well as the cytokine profile in this area are important for pregnancy outcome. This was clearly portrayed by Co *et al.* [153] who have shown that fetal trophoblasts are well-tolerated by the decidual NK cells through the macrophage-dependent production of TGF-β. If macrophages are lacking or an anti-TGF-β1 neutralizing antibody is used to deplete TGF-β1 from the equation, NK cells will be enabled to kill trophoblasts by lysing them. Therefore, the fact that in placentas obtained from preeclamptic patients, the TGF-β signaling pathway is down-regulated [148], together with the observations that in these placentas, the population of macrophage cells is increased [151], raise the possibility of a reasonable immune rejection of the placenta by altered decidual cells in preeclampsia. Notably, the early dominant immune population in the feto-maternal interface is based on NK cells and macrophages, while T and B lymphocytes are rather rare, highlighting the importance of the innate response type of cells during placentation [154]. Primarily, the NK cells' cytokine repertoire includes TNF-α, granulocyte–macrophage colony stimulating factor (CM-CSF), colony-stimulating factor 1 (CSF-1), IL-12, IL-15, IL-18, and especially IFN-γ [146], while that of macrophages includes TGF-β as well as IL-1, TNF-α, and IL-6.

Thus, it is clear that deficient placentation is detrimental for the overall pregnancy process. Indeed, it has been demonstrated that poor stromal cell proliferation/differentiation, reflected as reduced decidualization, may lead to preeclampsia or other pathological outcomes [134]. Another interesting feature of the placentas obtained from preeclamptic women is the observation of a sexually dimorphic level of inflammation. Indeed, in preeclamptic pregnancies, the placentas of male fetuses were associated with a significantly higher expression of inflammation, hypoxia, and apoptosis factors, but a reduced expression of pro-angiogenic markers compared to placentas of female fetuses [155].

## 6. Effects of Calcitriol upon Inflammatory Cytokines in Human Placenta

It has been documented by Bowen *et al.* [156] that human placenta and extra-placental membranes are able to produce several cytokines, including those involved in inflammatory process. In our laboratory, we have been interested in studying the following pro-inflammatory cytokines IL-6, INF-γ, and TNF-α, as well as the anti-inflammatory cytokine IL-10 in cultured trophoblast cells [93,101,102]. We have chosen these cytokines since they have been the most studied in preeclampsia, as mentioned above. In order to mimic a pro-inflammatory condition, trophoblast cells were treated with TNF-α. TNF-α stimulated IL-6, INF-γ, and its own expression more than three-fold over non-stimulated cells. The presence of calcitriol resulted in a dose-dependent inhibition of the expression of these three cytokines. The co-incubation of TEI-9647, a specific VDR antagonist, prevented the inhibitory effect of calcitriol upon the expression of these cytokines, indicating that this effect was VDR-dependent. Interestingly, the highest calcitriol concentration tested resulted in decreased gene expression with values similar to those observed in control experiments [93]. Using cultured trophoblast cells obtained from preeclamptic women, we have observed that basal gene expression of IL-6 and TNF-α decreased in a time-dependent manner [102]. We have demonstrated that IL-6, INF-γ, and TNF-α were significantly higher in placental cell cultures from preeclamptic women as compared with cells obtained from normotensive pregnant women. Interestingly, calcitriol treatment resulted in a dose-dependent down-regulation of these pro-inflammatory cytokines. It is of note that INF-γ was not detected in the culture media. However, calcitriol treatment resulted in a decreased concentration of IL-6, INF-γ, and TNF-α, both at the mRNA and protein levels [102]. On the other hand, we found that calcitriol inhibited the mRNA expression and protein levels of IL-10 in cultures obtained from normotensive pregnant women. The gene expression of IL-10 has been found significantly lower in placental cells obtained from preeclamptic women [101]. Interestingly, IL-10 decreases gene expression of β-defensins and cathelicidin, while calcitriol treatment stimulates the expression of these two antimicrobial peptides in cultured trophoblast cells [76]. In this respect, the inhibitory effect of calcitriol upon IL-10 expression could result in a more robust innate immune response in the human placenta.

## 7. Conclusions

The present review brings out information about vitamin D metabolism in pregnancy and its relation with inflammatory processes in preeclampsia. Indeed, maternal and placental calcitriol levels are low in preeclampsia. This alteration may be related to several dysfunctions at the maternal and placental compartments, including the deregulation of the immune system, which is characterized by high pro-inflammatory cytokine levels in preeclampsia. Since several studies showed that maternal calcidiol levels are low in pregnant women who subsequently developed preeclampsia, vitamin D supplementation has been suggested in order to reduce the incidence of preeclampsia.
